# A report from the NIHR UK working group on remote trial delivery for the COVID-19 pandemic and beyond

**DOI:** 10.1186/s13063-021-05880-8

**Published:** 2021-12-11

**Authors:** Jane A. H. Masoli, Kim Down, Gary Nestor, Sharon Hudson, John T. O’Brien, James D. Williamson, Carolyn A. Young, Camille Carroll, on behalf of the NIHR Remote Trial Delivery Working Group

**Affiliations:** 1 Royal Devon and Exeter NHS Foundation Trust, Exeter, UK; 2 College of Medicine and Health, University of Exeter, Exeter, UK; 3 NIHR Clinical Research Network Cluster E, Campus for Ageing and Vitality, Newcastle University, Newcastle, UK; 4 Cornwall Partnership NHS Foundation Trust, Bodmin, UK; 5 Department of Psychiatry, University of Cambridge School of Clinical Medicine, Cambridge, UK; 6 Cambridgeshire and Peterborough NHS Foundation Trust, Cambridge, UK; 7 National Institute for Health Research, Leeds, UK; 8 Walton Centre NHS Foundation Trust, Liverpool, UK; 9 University of Liverpool, Liverpool, UK; 10 University of Plymouth, Plymouth, UK

**Keywords:** Remote trial delivery, Virtual research, Trial methodology, Remote research

## Abstract

**Background:**

Prior to the COVID-19 pandemic, the majority of clinical trial activity took place face to face within clinical or research units. The COVID-19 pandemic resulted in a significant shift towards trial delivery without in-person face-to-face contact or “Remote Trial Delivery”. The National Institute of Health Research (NIHR) assembled a Remote Trial Delivery Working Group to consider challenges and enablers to this major change in clinical trial delivery and to provide a toolkit for researchers to support the transition to remote delivery.

**Methods:**

The NIHR Remote Trial Delivery Working Group evaluated five key domains of the trial delivery pathway: participant factors, recruitment, intervention delivery, outcome measurement and quality assurance. Independent surveys were disseminated to research professionals, and patients and carers, to ascertain benefits, challenges, pitfalls, enablers and examples of good practice in Remote Trial Delivery. A toolkit was constructed to support researchers, funders and governance structures in moving towards Remote Trial Delivery. The toolkit comprises a website encompassing the key principles of Remote Trial Delivery, and a repository of best practice examples and questions to guide research teams.

**Results:**

The patient and carer survey received 47 respondents, 34 of whom were patients and 13 of whom were carers. The professional survey had 115 examples of remote trial delivery practice entered from across England. Key potential benefits included broader reach and inclusivity, the ability for standardisation and centralisation, and increased efficiency and patient/carer convenience. Challenges included the potential exclusion of participants lacking connectivity or digital skills, the lack of digitally skilled workforce and appropriate infrastructure, and validation requirements. Five key principles of Remote Trial Delivery were proposed: national research standards, inclusivity, validity, cost-effectiveness and evaluation of new methodologies.

**Conclusions:**

The rapid changes towards Remote Trial Delivery catalysed by the COVID-19 pandemic could lead to sustained change in clinical trial delivery. The NIHR Remote Trial Delivery Working Group provide a toolkit for researchers recommending five key principles of Remote Trial Delivery and providing examples of enablers.

**Supplementary Information:**

The online version contains supplementary material available at 10.1186/s13063-021-05880-8.

## Background

Even before the COVID-19 pandemic, the traditional model of clinical trial delivery confined to the research unit was starting to slowly change. Delivery of clinical trials outside the hospital or research unit reduces some of the barriers to participation in clinical research and is aligned with the National Institute of Health Research (NIHR) project Innovations in Clinical Trial Design and Delivery for the Under-served (INCLUDE) [1], aiming towards greater inclusivity in clinical research. Restricting trials to those with the time, fitness and willingness to travel to research settings restricts potential participants, introducing bias. This can be due to distance from the research site, reduced mobility or ill health, challenges with transport or external commitments such as work or caring responsibilities [2, 3]. Delivery of clinical trials remotely for those who could not attend these settings had been slowly increasing prior to the start of the COVID-19 pandemic. Remote formats included telephone follow-up, online patient recruitment and interventions [4, 5]. Initiatives such as Trials@Home were aiming to develop standards and make recommendations on remote delivery and decentralisation of clinical trials [5].

The COVID-19 pandemic necessitated rapid, radical changes in clinical trial delivery. There was an immediate risk to public health, vulnerable groups were shielded, healthier participants were wary of hospital environments and the use of research facilities was restricted to minimise co-working and person-to-person contact. COVID-19 clinical trial activity was prioritised in many countries, including the UK, while other studies were largely paused to make way for urgent public health studies and to minimise potential COVID-19 exposure and spread in the research setting. The European Medicines Agency (EMA) [6] and the US Food and Drug Administration (FDA) [7] issued rapid guidance on assuring participant safety, maintaining compliance with good clinical practice (GCP) and minimising the risk to trial integrity. Worldwide, clinical researchers were forced to reconsider and restructure clinical trial protocols to make them workable and safe during a pandemic. This meant transferring as many aspects of the clinical trial as was considered safe and valid to remote delivery to minimise travel and potential exposure. However, not all sites or investigators had sufficient knowledge, facilities, infrastructure and familiarity of remote processes to make these considerable system changes, and disparate research groups met and addressed challenges independently.

The NIHR Clinical Research Network, which supports participation in high-quality research across England, formed a Remote Trial Delivery Working Group to consider the impact of a transition to remote trial delivery at each stage of the clinical trial process. The aim of this Working Group was to facilitate sharing of knowledge and best practice, addressing participant safety and concerns were central to the work. We aimed to collate information on practice of remote delivery of clinical trials, in the context of rapid pandemic-related system change. We also aimed to establish key principles of Remote Trial Delivery and to produce a toolkit to address potential challenges and provide examples of good practice for clinical researchers, funders and governance agencies to use when planning remote delivery of clinical trials. While the focus of the group was on clinical trials (i.e. studies with an intervention), findings from the toolkit may be equally applicable to many other clinical and non-clinical studies that have had to adapt to remote delivery.

## Methods

A national Remote Trial Delivery Working Group was formed by the NIHR in July 2020. Stakeholders included patient and public representatives; colleagues from across the clinical research network including from business development and marketing, research delivery and specialties such as ageing, genetics, neurological disorders and dementia; and colleagues from the industry.

### Defining remote trial delivery

The initial approach of the group was to establish a consensus definition of Remote Trial Delivery for the purpose of the work. We agreed that “Remote Trial Delivery” should include all activities related to clinical trial delivery undertaken without in-person, face-to-face contact.

### The trial delivery pathway

**Fig. 1:**
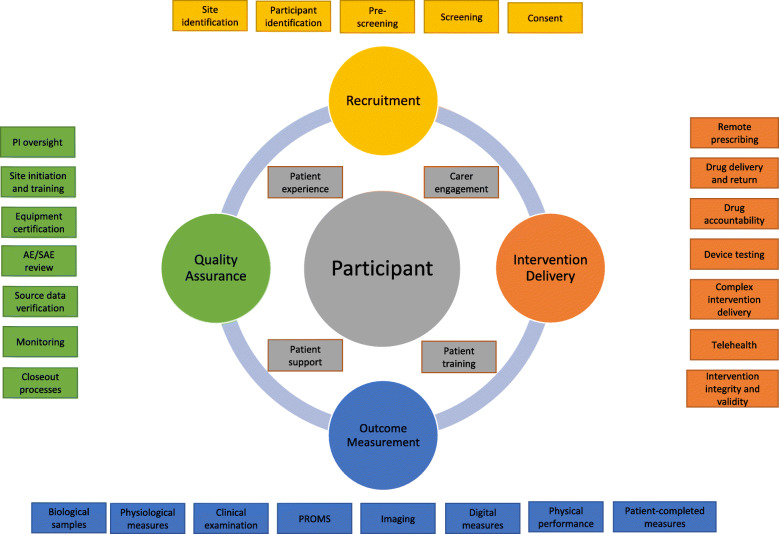
Key domains of the clinical trial delivery

We formed 5 subgroups based on the key domains of the trial delivery pathway: participant factors, recruitment, intervention delivery, outcome measurement and quality assurance, as detailed in Fig. 1.

**Fig. 2:**
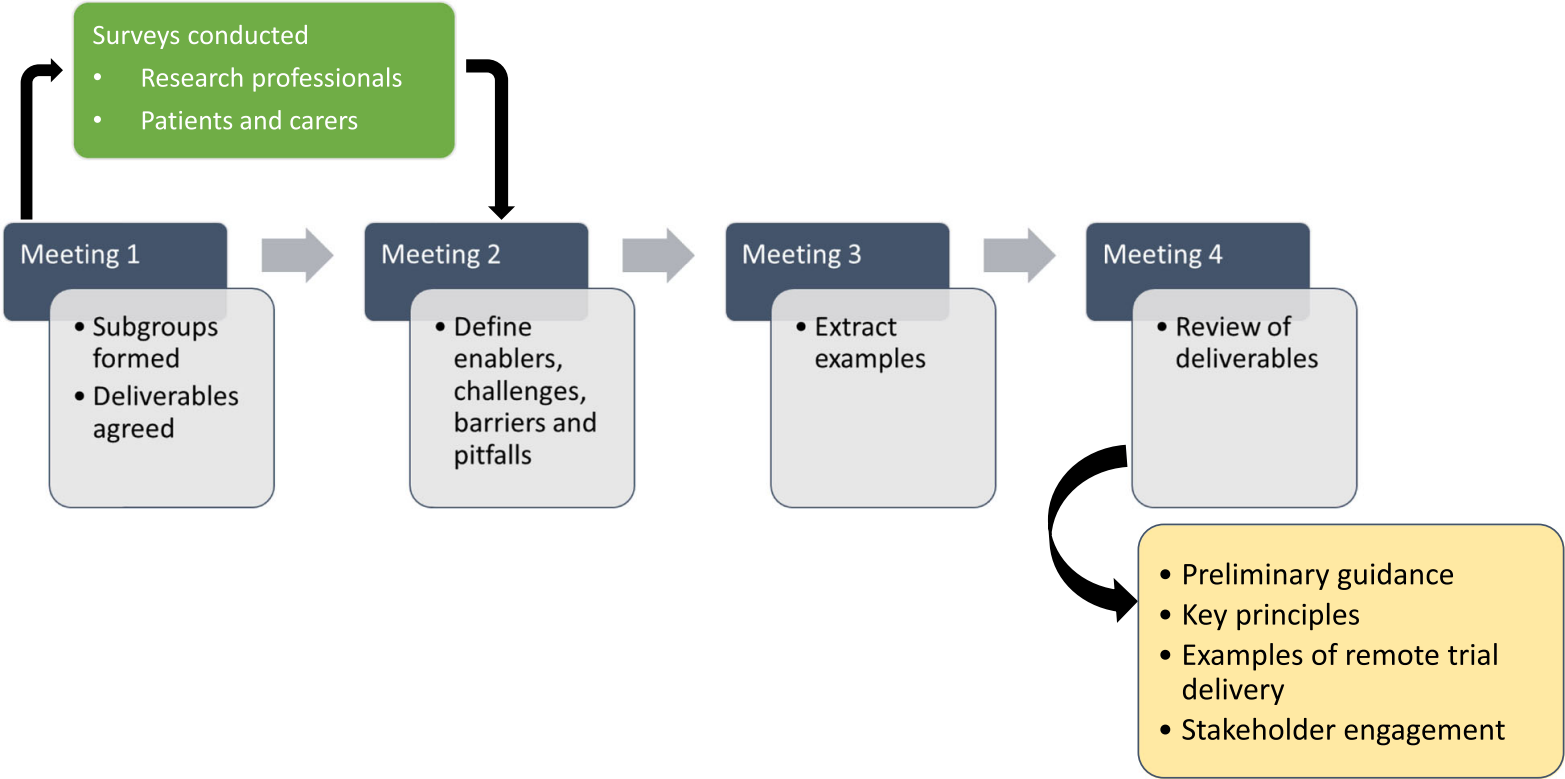
Working group work process between September 2020 and December 2020, with deliverables refined January to March 2021

In order to establish the potential benefits and pitfalls of Remote Trial Delivery, as well as challenges and enablers, each subgroup extracted examples from the two surveys outlined below, as well as reviewing NIHR case studies on restarting research [8] and pragmatic searching for additional studies that provided examples of remote delivery pertinent to that specific domain of the clinical trial pathway. The accumulated evidence was then streamlined into factors affecting participant experience, infrastructure and processes, and assessments and interventions. The key principles of Remote Trial Delivery were established through initial Working Group discussion with an iterative approach following subgroup refinement of existing practice within their domain (Fig. 2).

### Surveys

We designed two surveys that were disseminated concurrently in September 2020 to capture information on remote delivery across the clinical trial pathway (Additional file 1). Both surveys were implemented in order to ascertain challenges, enablers and examples of good practice, the first from participants and carers and the second from researchers and research delivery teams. Researchers were also asked to identify the greatest area of need in order to inform remote trial delivery. The surveys were created in Google forms with anonymised data capture and hosted by the NIHR. The researcher surveys were disseminated through national and regional Clinical Research Network newsletters, as well as through the cluster offices to their respective specialty leads to share wherever possible.

The participant and carer survey was co-developed with the NIHR patient and public involvement (PPI) representatives and was circulated via the NIHR PPI network and social media, dissemination via NIHR CRN networks and via the Association of Medical Charities to their members. The surveys were available from September to November 2020.

### Development of the NIHR remote trial delivery toolkit

We used the results from the methods detailed above to develop a toolkit for researchers seeking to make the transition to Remote Trial Delivery. A website was developed and launched in March 2021 [9], hosted by the NIHR, which encompasses the key principles of Remote Trial Delivery, and a repository of best practice examples, which were collated by this Working Group. In addition, the website includes questions to guide research teams in designing and delivering remote trials (Table 1).

**Table 1:** Questions to guide research teams in designing and delivering remote trials

1. Do you need to change the way in which you identify potential participants? Has the clinical care pathway changed?	
2. What are the infrastructure requirements for the proposed method of delivery (hardware, software, connectivity)? Are these supported by your organisation and available to potential participants?	
3. Will Remote Trial Delivery introduce bias? If so, how can this be mitigated?	
4. Are you able to provide participants with guidance and support in using the remote methods?	
5. How will you ensure participants feel supported and have the opportunity to ask questions?	
6. What training provision is there for your team and participants in any new methodologies?	
7. Are the interventions, assessments and outcome measures validated for the planned use?	
8. Will some aspects of trial delivery be more difficult (e.g. some safety assessments)? How will this be mitigated?	
9. Are there any concerns regarding data security as a result of remote delivery?	
10. Do you need to adapt your processes to ensure data integrity?	
11. What additional processes for PI oversight do you need to develop?	

## Results

### Patient and carer survey

There were 47 respondents to the patient and carer survey, 34 of whom were patients and 13 of whom were carers. Forty-five respondents were from across England (the area covered by the NIHR); 2 were from Wales. All adult age groups were represented, including eight aged over 75 years. Thirty-four (72%) were women. Forty-three respondents were white; 2 were mixed ethnicity and 2 non-declared. Thirty-two (68%) had had experience of remote delivery of at least one aspect of a clinical trial (online or by post or telephone). The patients and carers were asked to consider the advantages and disadvantages of Remote Trial Delivery and to cite any personal examples. From this, we were able to develop themes of advantages and disadvantages of Remote Trial Delivery from the participant perspective, and we combined these themes with the results of the professional survey and pragmatic searches. The key themes of potential advantages and disadvantages of Remote Trial Delivery from the patient and carer perspective supported by examples of survey extracts are summarised in Table 2.

**Table 2:** Key themes of (a) advantages and (b) disadvantages of remote trial delivery from the patient and carer perspective, with example extracts from the survey

**(a) Advantages of remote trial delivery**	
Time	“can complete in own time” “better for working age participants as saves time” “less time waiting around” “have time to consider answers” “no time pressures” “less disruption to carers” “don’t have to rearrange childcare”
Feeling safe	“less anxiety for him” “reduced risk of COVID infection” “feeling of safety not going into hospital” “relaxed when dealing with researchers” “in my own comfort zone” “less stress about the appointment so anxiety levels lower”
Travel and transport	“convenience of not travelling” “no parking problems” “no stress getting to appointments on time” “no travel prep or stress so clearer results for researchers (meaning assessment is not of the travel stress but of his actual condition)”
Accessibility	“being able to join in when there may not be much research happening nearby” “being able to do the study anywhere in the country” “rapid response to any problems arising” “good for people who can’t go out” “can still contribute” “can take part 24/7”
**(b) Disadvantages of remote trial delivery**	
Inclusivity	“may exclude certain groups in the population” “patient participants rarely representative of all social & cultural groups”
IT	“some glitches” “accessing the website wasn’t easy” “wifi is terrible (rural)” “not used to technology”
Communication	“(missed) seeing peoples’ full body language” “difficult to hear” “it needs to be easy enough to understand and follow remotely” “you don’t get contact…chat and fun with the nurses”
Validity	“not sure how the assessment scores compared to those face to face” “unable to collect most of the study outcomes” “the telephone follow-up was perfunctory” “questionnaires repeated often – no change to report – difficult to be consistent” “If treatment or medication was being assessed I’d prefer face to face contact”
Support	“difficult to get help with tasks” “not having anyone to contact in the event of a problem” “I didn’t feel particularly supported” “any questions arising had to be raised at a later date” “lack of potential support” “nobody to answer questions or provide help in case of problems”
Value	“feeling like a number in a study so not feeling valued as a participant” “impersonal”

### Professional survey

The survey included 115 examples of remote trial delivery practice entered by respondents from 13 of the 15 local CRN regions in England. Respondents represented primary and secondary care research, with a broad reach across 28 clinical specialties. The research professionals identified patient experience as the aspect of Remote Trial Delivery most in need of further evidence to inform future successful remote delivery of studies, followed by outcome measures and quality assurance, and then recruitment, with intervention ranked as having the least need. The themes of identified uncertainties, knowledge gaps and developments required are summarised in Table 3.

**Table 3:** Uncertainties or knowledge gaps identified by research professionals and developments required to facilitate effective Remote Trial Delivery

Identified uncertainties, knowledge gaps and developments required	
Technology	Specific trial elements will need technological input: • Electronic site files • Integration between electronic systems • Digital consent • Data security, storage and access • Digital signatures
Training and skill development	Required for both research teams and participants
Communication	Digital communication will need to be facilitated: • With participants • Between participants • Between researchers • With the NHS, including electronic patient records and communication with healthcare workers
Validity	The validity of specific elements of remote delivery will need to be established, including: • The consent process • Interventions • Study measures
Participant factors	It will be important to create an evidence base to improve understanding of aspects relevant to patients and carers, including: • Safety • Acceptability • Bias/exclusion • Support • Impact on recruitment • Valid consent • Retention in trials • Return to future trials
Governance	Governance procedures will be required to ensure: • Standardisation • Quality • Data security • Sponsor and regulator support for remote processes
Resources	A comparison of the resources required for remote delivery versus face to face should include: • Time compared to “traditional” model • Cost compared to “traditional” model • “Buy-in” from sponsors

### Benefits, pitfalls, challenges and enablers of remote trial delivery across the research pathway

The working group identified key benefits, pitfalls, challenges and enablers to Remote Trial Delivery across the trial pathway utilising examples from the two surveys combined with those extracted from the literature (Table 4). In order to guide sites and researchers in thinking through the challenges, a series of questions was developed and posted within the toolkit on the website, together with case study illustrations of where particular processes have been successfully deployed.

**Table 4:** Benefits, pitfalls, challenges and enablers of Remote Trial Delivery

	Benefits	Pitfalls	Challenges	Enablers
**Participant experience**	Broader reach and inclusivity (in IT-enabled groups); increased research opportunity Flexibility in study delivery, increased convenience (e.g. not having to arrange transport and parking at the study site) Reduced infection risk Socially or geographically targeted recruitment	The impression of being “alone” (unsupported) Reduced contact with the study team might impact on retention Potential for non-compliance (intentional and unintentional) Potential for bias (e.g. due to limitations in remote communication e.g. computer literacy (digital divide, age-related/socioeconomic), literacy, audio-visual impairments) Reduced ability to ask questions or seek clarification Loss of non-verbal communication/difficulty with holistic assessment	Digital infrastructure and literacy Pathways to approach potential participants/care partners Adaptations required for inclusivity—e.g. hearing, visual or cognitive impairment Failure to maintain participant engagement Communication, including post-trial	Digital infrastructure and literacy Provision of guidance and support with technology and trouble-shooting Ensuring participant well-prepared and followed up regularly Provision of guidance and training on protocol adherence Peer support opportunities for participants (e.g. virtual coffee mornings) Safety net of regular video contact Clear route for communicating with the study team
**Infrastructure and processes**	Can be standardised/centralised Improved efficiency	Threat to privacy/confidentiality Data security Protocol compliance more difficult to assess	Outdated NHS IT systems (NHS IT—National Health Service Information Technology) Quality control/standardisation harder to ensure in diverse environments More resource-demanding Increased preparation time for remote monitoring Variation in information governance processes and standards Maintenance of essential documentation	Electronic patient/medical record/HSCN (Health and Social Care Network) Standardised processes (e.g. FHIR Fast Healthcare Interoperability Resources) Approved e-consent process Central coordination Consent and ethics approval in place for remote monitoring Flexible mindset within organisations Experienced workforce with appropriate skills
**Assessments and interventions**	Greater ecological validity Potentially greater clinical validity than “snap-shot” in-clinic assessments Captured with greater granularity Reduced data capture error Reduced risk of research fraud More efficient data analysis	May not be validated Potential for reduced safety assessments Potential for increased heterogeneity within measurements	Unsupervised environment for delivery Requirement for demonstration of validity/equivalence Need to be acceptable to regulators Lack of staff familiarity Should be feasible and acceptable to patients Not all interventions can be delivered remotely	Developed and validated for remote delivery Equivalence to traditional measure demonstrated

### Key principles of remote trial delivery

The NIHR Remote Trial Delivery Working Group refined five key principles of Remote Trial Delivery, supported by the results of the surveys conducted in participants and carers and research professionals.

These principles are:

1. National standards for trial delivery best practice should apply
2. Inclusivity should be maximised
3. Measures and processes should be validated
4. Trial delivery should be cost-effective
5. New methodologies should be robustly evaluated

## Discussion

The COVID-19 pandemic had an immediate impact on clinical trial delivery and provided an opportunity for rapid, systemic change. There was an immediate removal of former barriers to restructuring towards a remote delivery approach. The Remote Trial Delivery Working Group recommended five key principles of Remote Trial Delivery and developed a toolkit to guide researchers, underpinned by a targeted literature review and surveys of research professionals, participants and carers. As well as the key principles and toolkit, we have assembled a repository of good practice examples available on our website.

The speed of change away from face-to-face delivery without clear methods and signposting undoubtedly led to rapid development of innovations in research planning and delivery, from which valuable lessons can be learned. The wider societal shift to remote working has provided considerable upskilling in digital communications and introduced innovative, technology-supported ways of working, reducing the need for physical meetings. Participants and researchers described significant benefits of remote delivery of some aspects of clinical trials in addition to reduced COVID-19 infection risk. For example, broader reach and inclusivity in IT enabled groups and improved efficiency and flexibility for both research teams and participants. In addition, once the infrastructure is sufficient to support remote trial delivery with centralisation of processes, Remote Trial Delivery is likely to reduce the costs and environmental impact of clinical research.

However, the benefits of Remote Trial Delivery are not universal, and significant knowledge gaps remain. Importantly, Remote Trial Delivery most frequently involves the use of online platforms, or applications, which can introduce bias in participant recruitment and ability to participate. The “digital divide” is the gap between people in society who have full access or skills to enable the use of digital technologies, such as the Internet and computers, and those who do not [10]. In the UK, the main factors influencing the digital divide are age, region, socioeconomic status and whether a person has a disability [10]. Other important factors to consider with facilitating accessibility are language barriers and the requirements of under-served groups. Following COVID-19, research will be needed to establish how the wider use of the Internet and video conferencing has altered access and familiarity with online communication. This should be considered when planning and delivering clinical trials. It is also essential to have a digitally literate and competent workforce to deliver clinical trials remotely, for all aspects of clinical trial delivery including ensuring a good quality patient experience. This will require the right platforms, processes and infrastructure, for example, access to electronic healthcare records for remote monitoring, the ability to store, manage and transfer electronic documents and appropriate electronic consent platforms.

The research professionals’ survey respondents did not consider the intervention as a leading trial component in need of further evidence in order to provide Remote Trial Delivery. In some trials—for example, drug trials—the intervention delivery may not be significantly altered by remote delivery, which may be why this was not considered a higher priority. However, a major potential limitation of Remote Trial Delivery is that there is currently insufficient evidence to support the validity of many specific interventions and assessments when delivered remotely and more research is required. Equally, quality assurance processes need to continue to be rigorous, including monitoring of data integrity and oversight processes sufficient to meet the appropriately high standards of regulators. Providing sufficiently rigorous regulation while enabling innovation is a further challenge. Standards for metadata to support digital health technologies in clinical research [11] and frameworks for Biometric Monitoring Technologies [12] have been proposed. However, there is no formal standard that is widely accepted. In addition, the participant voice in not feeling valued in the same way as in previous trial experiences or having the same personal interactions with the research team may impact on the recognised benefit of being enrolled in clinical trials regardless of the intervention group, as well as reducing willingness to participate and retention in follow-up.

The potential advantages and disadvantages of Remote Trial Delivery that we have outlined are in keeping with previous reports. The Trials@Home group released a first set of recommendations for remote delivery of clinical trials in August 2020 [13], based on a systematic literature review and a consortium of public and private partners. The themes that they described of potential advantages and limitations correlate well with our findings. There is a growing industry drive to decentralisation and remote delivery of clinical trials citing improved participation opportunity, trial efficiency and quality, with some of the transformation and decentralisation drives receiving cross-sector support including government, patient groups, sponsors, professional societies and academic institutions [14].

### Strengths

This NIHR Remote Trial Delivery Working Group formed from a broad range of national stakeholders has delivered a consensus opinion on the key principles of Remote Trial Delivery and a toolkit for researchers to use when planning remote delivery. This work included patients and participants as key members of the Working Group and designed and implemented separate surveys for participants and research professionals. This adhered to the goal of keeping the participant as the central focus of this work. This approach enabled us to present the participant views, as well as the overall themes of potential advantages and disadvantages of Remote Trial Delivery.

### Limitations

Similar to the report from the INCLUDE group [1], the Remote Trial Delivery Working Group conducted this work as a focussed, time-limited project. This work needed to be expedited to provide a toolkit and recommendations at a crucial period of service re-design in clinical trial delivery due to the COVID-19 pandemic. This led to some compromise in the approach. Firstly, the literature was not reviewed using a prespecified protocol or a systematic search. Secondly, surveys, while widely circulated via national networks, were time limited and targeted. We recognise that the participants and carers surveyed represent those on the privileged side of the digital divide and are not a representative sample of the overall participant population. Almost all respondents to our surveys were from England; other localities may experience different challenges and enablers which we were not able to explore. In addition, almost all of the respondents to our patient/carer survey were white, limiting our ability to explore aspects of relevance in other ethnic groups, such as trust, accessibility and language. As these surveys were conducted during the acute phase of the COVID-19 pandemic, they capture a period in which the majority of experience may relate to COVID-19 studies or work-arounds to maintain studies that were unable to continue if not remotely delivered. Ongoing work is required to understand remote trial delivery when clinical trials are less restricted.

### Future steps

The NIHR is supporting the development of complex and innovative trial designs, such as those with virtual, decentralised or siteless trials [15]. However, further research is required across the research pathway to establish the scientific robustness of Remote Trial Delivery, as well as ensuring that the quality of the participant experience is maintained. This should include validation studies for specific methods and outcome measures, alongside work to assure data quality across the trial pathway. This should be tailored to whether the measure is an existing measurement applied to a different setting e.g. home setting replication, or whether it is a new measure incorporating digital measurement or a novel concept or end point.

We have identified knowledge gaps and developments required in order to optimise the potential for Remote Trial Delivery. These include changes in processes, infrastructure and workforce, especially with technology, data and training. Clinical trial delivery will need to evolve to clinical care pathways of the future [16], in accordance with the NHS Long Term Plan [17] in which digitally enabled care will be mainstream. We need to ensure that we build the infrastructure and skills now to support the delivery of hybrid or fully remote clinical trials, building on the principles described.

## Conclusion

The transition towards Remote Trial Delivery was an unanticipated consequence of the COVID-19 pandemic. Remote Trial Delivery has potential benefits for research teams and participants, but the recommended five key principles (national research standards, inclusivity, validity, cost-effectiveness and evaluation of new methodologies) should be adhered to, to ensure scientific robustness and participant safety. Transitioning to Remote Trial Delivery requires adequate planning, and we have provided a toolkit to highlight potential challenges and barriers, with examples of enablers and further examples of best practice available on our website. In the medium to longer term, clinical trials are likely to operate in a spectrum from the traditional model to full remote delivery, with the majority of trials operating some components remotely. Clinical trial delivery has been impacted by COVID-19, but with it has come the opportunity for change and to retain positive practices in Remote Trial Delivery to be fit for purpose for the future.

## Supplementary Information

**Additional file 1.** Surveys.

## Data Availability

Surveys are included in the supplementary material. Data are available from the authors upon reasonable request.
