# Supplementary material for: A report from the NIHR UK working group on remote trial delivery for the COVID-19 pandemic and beyond
Source: Trials. 2021 Dec 11;22:911. doi: 10.1186/s13063-021-05880-8 (PMC8665850; doi:10.1186/s13063-021-05880-8)
Supplement: Supplementary file 1 — Additional file 1. Surveys. [file 13063_2021_5880_MOESM1_ESM.docx]

**Supplementary Material: Surveys**

*Surveys adapted into word document format for supplementary material.*

**Research Professionals Survey**

Remote Trial Delivery: Practice Examples

We are seeking your advice as clinical trial design and delivery experts to help answer some key questions as part of an important national project to inform future NIHR policy and procedures.
We are looking for examples from you of designing and delivering trials remotely. Specifically, examples of recruitment, outcome measures, intervention, patient experience, and quality assurance. We would be grateful if you could give us as much information as possible about any of the areas that you have experience in.
These examples are being collected in order to inform a position paper from the Remote Trial Working Group to the NIHR Clinical Research Network Senior Management Team and for possible publication.
We hope that some examples provided may be worked up by us further so that, following appropriate consent, they could be used as exemplars of best practice.
You are able to enter up to 5 examples on this form. If you would like to enter more, then please click the link at the end to relaunch the survey.

- Which Local Clinical Research Network do you work within?
- What is your specialty area?
- Examples of Remote Trial Delivery

This is an example of remote trial delivery relating to... *

Recruitment (patient identification/Initial approach/Pre-screening/Screening/Patient consent/Other:)

Intervention (Telehealth/Device Testing/Remote prescribing/Intervention integrity and validity/Complex intervention delivery/Drug accountability/Other:)

Quality Assurance (Closeout processes/Site initiation and training/Source data

verification/Monitoring visits/AE / SAE monitoring/Equipment certification/Other:)

Outcome Measures (Biological samples/Physiological measures/Clinical

examination/PROMS/Imaging/Digital Measures/Physical performance/Patient-completed measures/Cognition/Other:)

Patient Experience (Patient/carer engagement/Patient/carer support/Information provision

Patient training/Patient experience of research/Other:)

- Examples of Recruitment

What is your example?

Your answer

Please include a link to the study website, or a relevant publication, if available.

Your answer

Why have you chosen this example?

Your answer

What worked well, in terms of the approach?

Your answer

What didn't work well with this approach?

Your answer

Please provide any other comments or information.

Your answer

- Would you like to provide another example?  *

Yes

No

- How could remote trial delivery be improved?
- What are the main uncertainties where more evidence or knowledge is needed to facilitate effective remote trial delivery?
- Please rank the following in order of the greatest need/importance to acquire new evidence/ knowledge to inform the future successful delivery of remote studies

(most important to least important)

Recruitment

Quality Assurance

Outcome Measures

Intervention

Patient Experience

- Please explain your choice of response

**Patient and carer survey**

Running medical research studies remotely

The National Institute of Health Research (NIHR) are doing some work to look at the participant and carer experience of taking part in clinical research studies where, rather than a person having to attend a study visit in person, some or all of the study visits are delivered ‘remotely’ (ie – not in person, for example using post, telephone, email or video-link). COVID-19 has already led to lots of different ways of conducting study visits and we want to find out what is working well, what isn't working well and how we can improve studies in the future for participants. We would like you to complete the questionnaire below. The information you provide will be used to guide teams planning clinical research studies.

* Required

- Are you completing this form as a *

Participant in a clinical research study

Carer of someone participating in a clinical research study

- What is your age *
- What is your sex (as assigned at birth)

Female

Male

Prefer not to say

- What is your ethnic group?

Choose one option that best describes your ethnic group or background

- Which geographical region best represents where you live? *

1. North East and North Cumbria

2. North West Coast

3. Yorkshire and Humber

4. Greater Manchester

5. East Midlands

6. West Midlands

7. West of England

8. Thames Valley and South Midlands

9. Eastern

10. Kent/Surrey/Sussex

11. Wessex

12. South West Peninsula

13. North Thames

14. South London

15. North West London

Northern Ireland

Scotland

Wales

Other:bout clinical research studies

Taking part in a clinical research study involves learning about the study, being given information about it, discussing it with the research team and then deciding whether or not to take part. During the study, you will have various assessments and may have to perform certain tasks, for which you might require training. The study may involve taking a new medication or having a new form of therapy. We are interested in learning about your experience of all these aspects of clinical research studies, if they are delivered remotely.

- Are you involved in clinical research studies? *

Tick all that apply

I/someone I care for is currently enrolled in a medical research study

I/someone I care for has previously taken part in a medical research study

Other:

- Have you (or someone you care for) taken part in a clinical research study where some or all of the experience was ‘remote’, where some or all of the study visits were replaced with other means of collecting information (ie – not in person, for example using post, telephone, email or video-link)? *

Yes

No

Not sureur thoughts about remote clinical research studies

- What would you consider to be the top 3 ADVANTAGES to you or someone you care for joining a clinical research study run remotely?

Your answer

- What would you consider to be the top 3 DISADVANTAGES to you or someone you care for joining a clinical research study run remotely?

Your answer

- What would you consider to be the top 3 BARRIERS to you or someone you care for joining a clinical research study run remotely?

Your answer

- Do you think the overall experience for PARTICIPANTS would be better or worse if more medical research studies were delivered remotely? *

Better

1

2

3

4

5

Worse

Please briefly explain your answer to the previous question

Your answer

- Do you think the overall trial experience for CARERS would be better or worse if more medical research studies were delivered remotely *

Better

1

2

3

4

5

Worse

Please briefly explain your answer to the previous question

Your answer

- What could we do to improve the patient and carer experience in delivering studies remotely?

Your answer

Submit
